# Acute cardiac tamponade after Endostar treatment of non-small cell lung cancer: A case report

**DOI:** 10.1097/MD.0000000000038106

**Published:** 2024-05-10

**Authors:** Wanhui Dong, Mingxing Wang, Pei Zhu, Qingming Sun, Dezhen Wu

**Affiliations:** a Department of Medical Oncology, Lu’an Hospital Affiliated to Anhui University of Chinese Medicine, Anhui province, China.

**Keywords:** angiostatin, antiangiogenesis, cardiovascular events, case report, Endostar, pericardial tamponade

## Abstract

**Rationale:**

Recombinant human endostatin (Endostar) is extensively utilized in China for the clinical management of patients with driver gene-negative non-small cell lung cancer (NSCLC) at stage TNM IV. This report describes the case of a lung cancer patient treated exclusively with Endostar maintenance therapy, who experienced a rapid deterioration in respiratory function.

**Patient concerns:**

The case involved a patient with a pathologically confirmed squamous cell carcinoma of the left lung, treated in our department. Following 1 month of albumin-bound paclitaxel chemotherapy and localized radiotherapy for the left lung lesion, the patient initiated treatment with a single agent, Endostar 30mg, on October 19, 2021. The medication was administered via intravenous infusion over a 7 days.

**Diagnosis:**

On October 23, 2021, the patient exhibited symptoms of chest constriction, discomfort, coughing, and sputum production. By October 28, the patient presented with pronounced dyspnea and respiratory distress. An emergency CT scan detected pericardial tamponade and significant deviations in several blood parameters from pretreatment values.

**Interventions:**

Percardial puncture and catheter drainage were recommended as therapeutic intervention.

**Outcomes:**

Considering the patient advanced age, the patient and their family opted to refuse this medical procedure, leading to the patient unfortunate demise on November 2, 2021.

**Lessons:**

Medical professionals should remain vigilant for the potential, albeit rare, risk of Endostar inducing acute pericardial tamponade, a severe and potentially fatal complication.

## 1. Introduction

Primary lung cancer is a prevalent malignant tumor in clinical practice, with environmental exposures and tobacco use emerging as the leading etiological factors. Globocan2020 statistics reveal an incidence rate of approximately 2.2 million cases globally, with a mortality rate of 1.8 million cases.^[1]^ Between 2010 and 2017, a notable decrease in non-small cell lung cancer (NSCLC) incidence was observed in the United States, dropping from 46.4 to 40.9 per 100,000. Specifically, the incidence rate for stage IV NSCLC declined from 21.7 to 19.6 per 100,000, while stage I saw an increase from 10.8 to 13.2 per 100,000.^[2]^ This trend suggests a growing trend toward early detection and treatment. Conversely, in China, a developing nation, lung cancer incidence has surged over the past 3 decades, making it the predominant cause of cancer-related mortality.^[3]^ Regrettably, many patients only seek medical attention after symptom onset, often presenting with advanced disease.

Over the past decade, a paradigm shift has occurred in the management of advanced lung cancer, transitioning from a chemotherapy-centric approach to one that integrates targeted therapies, immunotherapy, and other precision medicine strategies. This evolution has yielded more definitive treatment outcomes for patients with diverse genetic and molecular profiles.^[4]^ Among the therapeutic advancements, anti-angiogenic drugs have become integral in NSCLC management due to their versatility in combination with various agents and their favorable safety profile.^[5,6]^ Notably, Endostar, a chemically modified recombinant human endostatin developed independently in China, has demonstrated superior stability and safety in treating a spectrum of solid tumors, including NSCLC, cervical cancer, and nasopharyngeal carcinoma.^[7–9]^

In comparison to platinum-based double-drug chemotherapy for NSCLC, the addition of Endostar to platinum-based double-drug chemotherapy has been associated with prolonged median survival and progression-free times (19 months: 14.3 months; 7.7 months: 5.4 months), alongside a favorable safety profile.^[10]^

Although numerous clinical studies have demonstrated the safety and effectiveness of combining Endostar with chemoradiotherapy,^[11,12]^ we recently observed a case of an elderly patient with lung squamous cell carcinoma who received single-drug Endostar maintenance therapy for the first time. He experienced chest tightness and then progressed to dyspnea on the fifth day of using Endostar. On the third day after completing of Endostar treatment, a computed tomography (CT) examination confirmed that the patient had developed acute pericardial tamponade. Although the instructions and clinical studies of Endostar have reported drug-related adverse cardiac reactions, only mild and moderate reactions that do not affect the use of the drug have been observed. There are no previous reports of severe adverse reactions, such as acute pericardial tamponade, in the literature.

## 2. Case presentation

**Figure 1.**
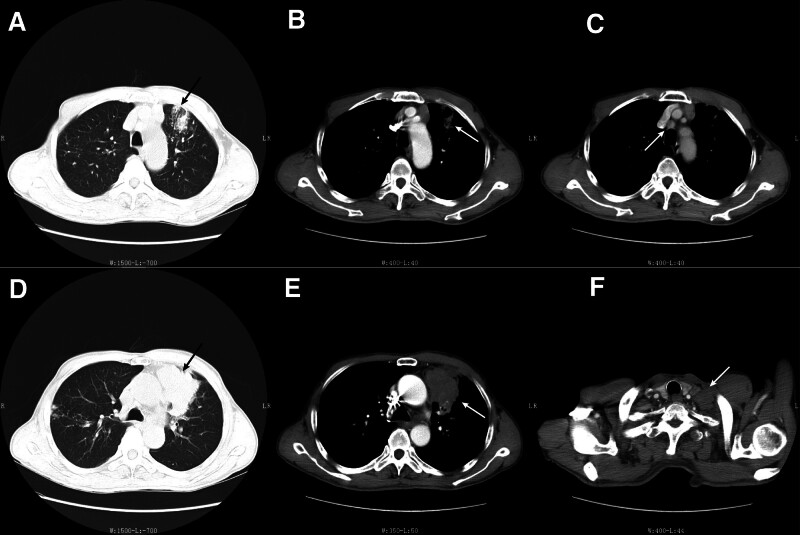
Endostar pre-use chest CT (plain + enhanced) (A) Pulmonary window at aortic arch level: patchy density shadow can be seen in the left lung; (B) Mediastinal window at aortic arch level: the left lung lesions with exudative changes; (C) Enlarged lymph nodes with marked enhancement can be seen in the mediastinum near the aortic arch; (D) Lung window at the level of tracheal bifurcation: Massive space-occupying lesion of left hilar mass; (E) Mediastinal window at the level of tracheal bifurcation: lumpy soft tissue-like density shadow with an unclear boundary and pleural adhesion. Inhomogeneous enhancement after enhanced scanning; (F) The left supraclavicular lymph node appeared enlarged and showed slight enhancement post-enhancement. CT = computed tomography.

**Figure 2.**
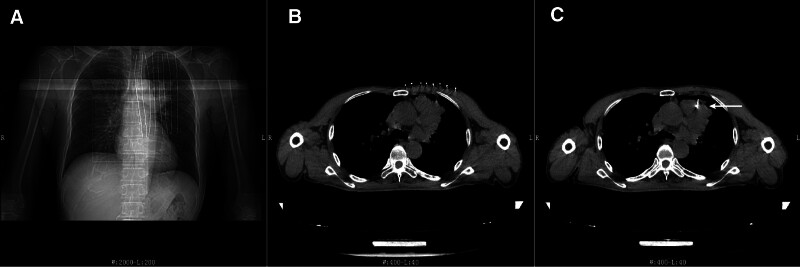
Puncture biopsy of the left lung using CT-guided localization for space-occupying lesion. The operator attaches a metal positioning wire to the patient body surface (A) and then uses tomographic images from the CT scan to determine the relative position of the left lung lesion to the body surface metal positioning wire, further planning the precise puncture path (B). Employing a 20G puncture biopsy needle (←), left lung lesions were biopsied under CT guidance (C). CT = computed tomography.

**Figure 3.**
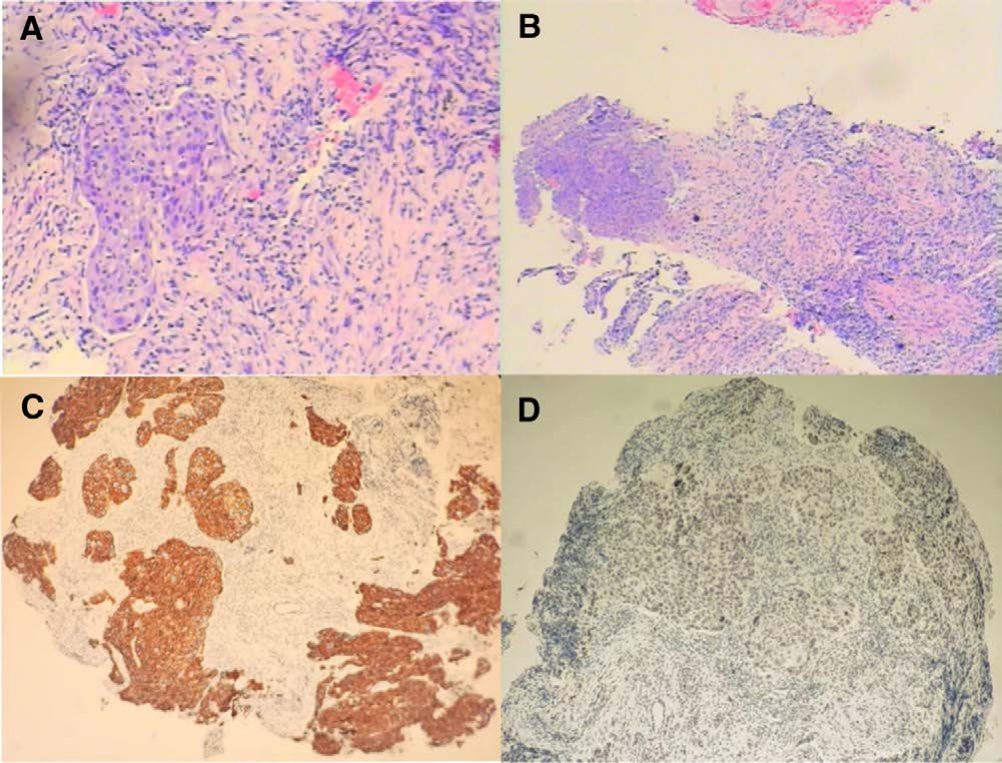
Pathological and immunohistochemical results of the puncture biopsy specimen from the left lung. (A-B) (left upper lung puncture tissue) non-small cell lung cancer (HE staining, magnification: 100×). (C-D) immunohistochemistry suggested squamous cell carcinoma. The tumor tissue tested positive for CK5/6 (C), and P40 (D).

During a routine annual examination, a 71-year-old male presented with space-occupying lesions in the left hilar region (Fig. 1). A subsequent needle biopsy on July 17, 2020 (Fig. 2), and pathological analysis (No. 2007573) with immunohistochemistry confirmed lung squamous cell carcinoma (Fig. 3), with positive CK5/6 and P40 expression. The patient, with a 3-year history of primary hypertension, managed his condition with indapamide 2.5 mg daily, maintaining a blood pressure of 140/90 mm Hg. He had no family history of genetic disorders and, with no financial income, reported a daily quality of life PS score of 2.

Subsequently, the patient underwent 4 cycles of chemotherapy with albumin-bound paclitaxel (Qilu Pharmaceutical, H20183378) at 300mg on day 1 and cisplatin injection 30 mg (Jiangsu Haosen, H20040813) from days 1 to 3. This regimen resulted in significant lesion reduction in the left lung, indicating partial remission. Owing to the patient advanced age, the family opted for surveillance every 3 months, rather than continuing chemotherapy. In July 2021, a CT scan revealed lesion progression, leading to conformal intensity-modulated radiation therapy for the left lung.

**Figure 4.**
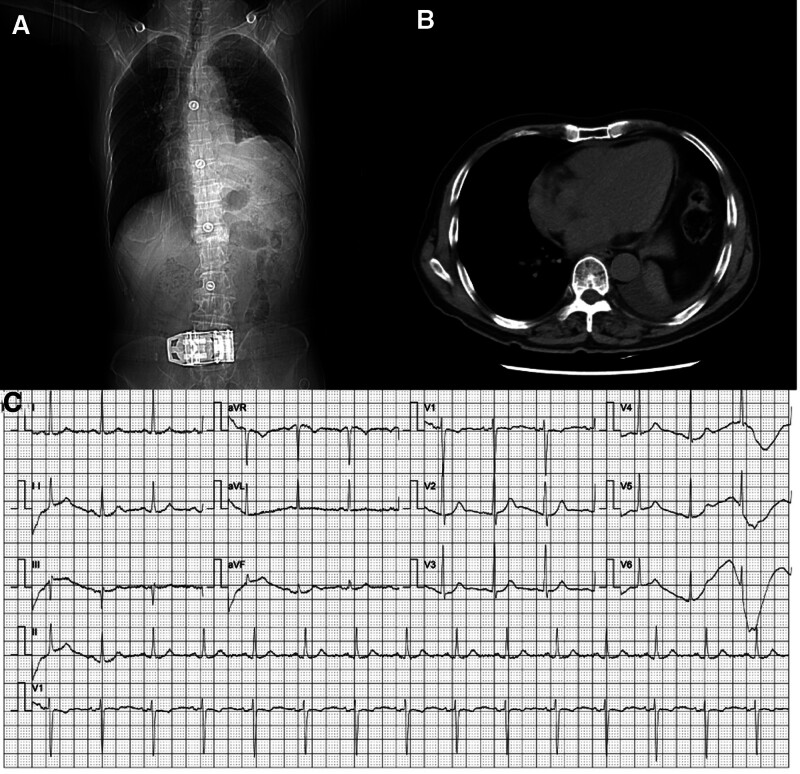
Baseline assessment of CT and ECG before the use of Endostar (A) The heart shadow enlarges. (B) There was a small amount of effusion in the pericardium and left thorax. (C) An ECG indicates sinus rhythm. CT = computed tomography, ECG = electrocardiogram.

**Figure 5.**
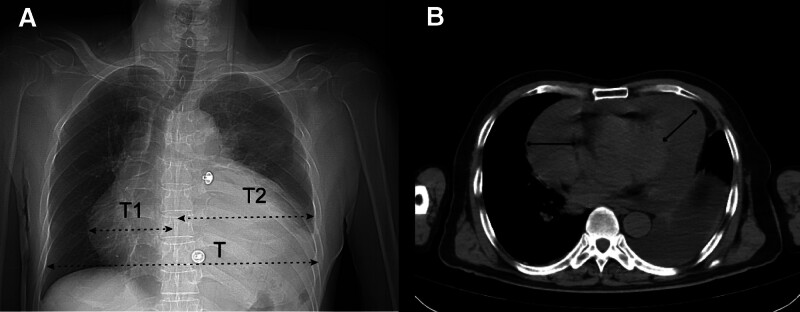
CT scan image on the 10th d after receiving Endostar treatment. (A) The cardiothoracic ratio was significantly larger than before treatment, exceeding 0.6. Cardiothoracic ratio = (T1 + T2)/T, where T1 represents the distance from the left heart margin to the midline, T2 represents the distance from the right heart margin to the midline, and T represents the maximum transverse diameter passing through the highest point of the right diaphragm. (B) A plain CT scan revealed acute pericardial tamponade and massive pericardial effusion, which were significantly increased compared to before the administration of Endostar. CT = computed tomography.

**Figure 6.**
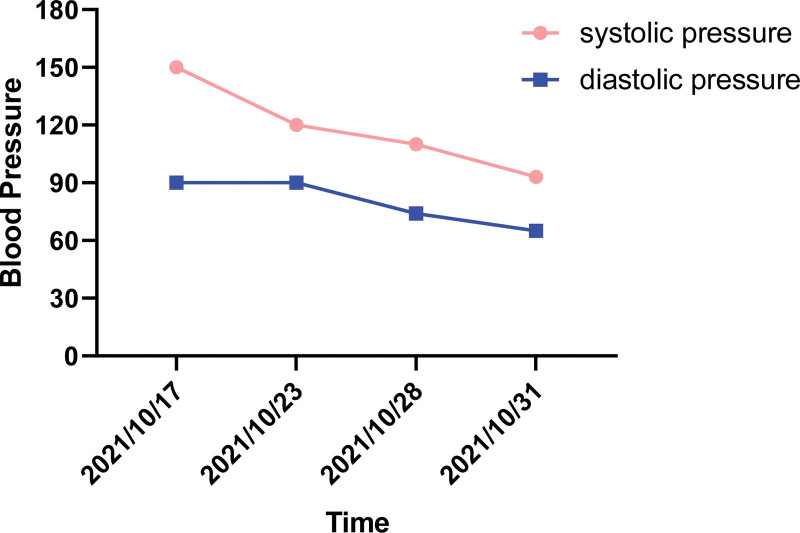
Line graph of blood pressure variations during the patient hospitalization Throughout the entire hospitalization process, both systolic and diastolic blood pressure showed a gradual decrease compared to the initial blood pressure upon admission.

**Figure 7.**
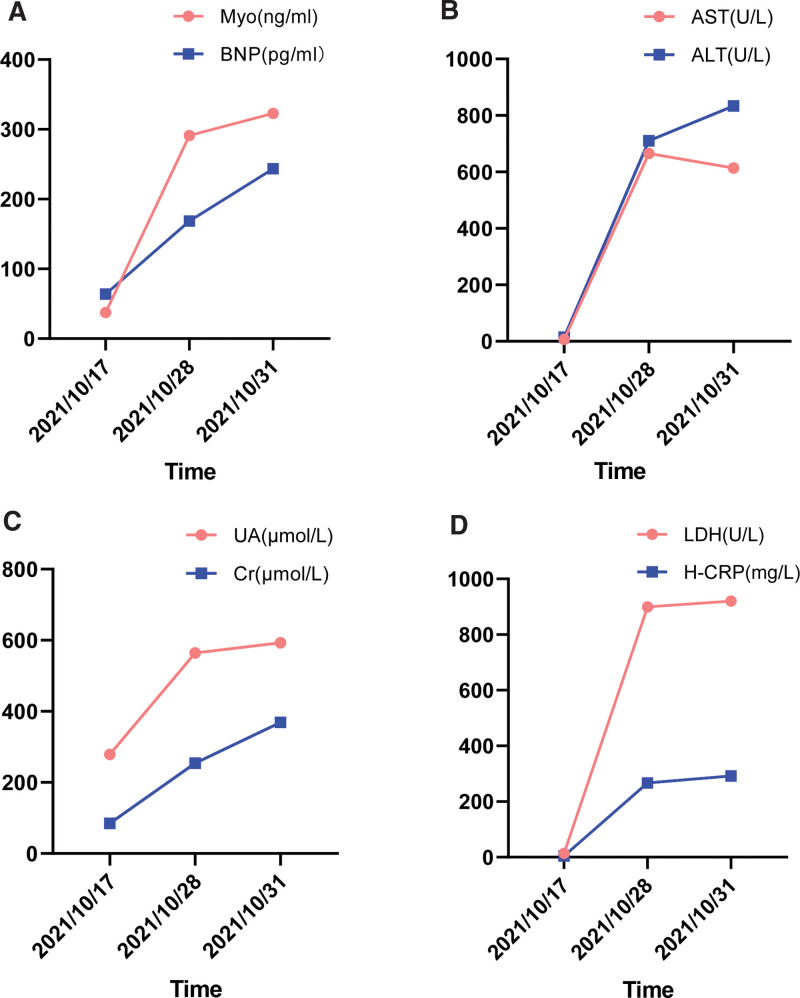
Trend of chemical indicators in the use of Endostar (A–D) During the Endostar treatment, the blood test results for Myo, BNP, Alt, AST, UA, Cr, LDH, and H-CRP showed varying degrees of increase. ALT = alanine transaminase, AST = aspartate aminotransferase, BNP = b-type natriuretic peptide, Cr = creatinine, H-CRP = high-sensitivity c-reactive protein, LDH = lactic dehydrogenase, Myo = myoglobin, UA = uricacid.

In October 2021, the patient was admitted for further maintenance therapy. Reviewed of the lung CT and electrocardiogram (Fig. 4) indicated an enlarged cardiac silhouette and minimal pericardial and left pleural effusion. Physical examination (PE) revealed that the breath sounds of both lungs were clear, there was no engorgement of the external jugular vein, and no clear contraindications to treatment. Considering the patient age and physical condition, and to mitigate the risk of post-radiotherapy pneumonia and bone marrow suppression, we adhered to NCCN guidelines (www.nccn.org/patients) and previous ASCO meeting research, administering a single agent,^[10]^ Endostar (Shandong Xiansheng pharmaceutical, s20050088), at a dosage of 30mg on October 19. The treatment was maintained via intravenous infusion for 7 days. On October 23, the patient experienced symptoms of chest tightness and discomfort, along with coughing and expectoration, which occurred on the fifth day of using Endostar. The patient has been diagnosed with squamous cell carcinoma of the left lung and has been experiencing a persistent cough, intermittent asthma, and stuffy symptoms throughout the course of the disease. During the PE, we observed no external jugular vein distension or arrhythmia. during the PE. Based on our clinical diagnosis, we still consider pulmonary dyspnea as the primary concern. Therefore, we have prescribed oxygen inhalation and Compound Glycyrrhiza tablets for orally treating cough. Despite this, in the days that followed, the patient experienced a significant worsening of breathlessness symptoms, characterized by orthopnea and mild cyanosis of the lips. Upon admission to the hospital, the patient underwent a chest CT on October 16. We observed a continuous worsening of the patient asthma and stuffy symptoms, along with distension of the patient external jugular vein. As a result, an urgent CT examination was performed on October 28, which indicated acute pericardial tamponade (Fig. 5). During the treatment, we observed a progressive decrease in blood pressure (Fig. 6) and a progressive increase in blood indicators such as b-type natriuretic peptide (Fig. 7). After cardiology consultation, pericardiocentesis was recommended to alleviate symptoms. However, the patient and his children decided against further interventions, resulting in discharge and, subsequently, the patient unfortunate demise on November 2, 2021.

## 3. Discussion

The concurrent use of endostatin with traditional chemotherapy and targeted therapy has shown enhanced clinical effectiveness and safety. The effectiveness of chemotherapy combined with endostatin in treating patients with advanced first-line NSCLC and negative driver genes was initially validated by the ECOG4599 study.^[13]^ Endostar, the first endostatin independently developed in China and introduced in 2006, primarily exerts its anti-tumor effects by downregulating vascular endothelial growth factor and its receptor. Additionally, it regulates the transforming growth factor-beta 1 and basic fibroblast growth factor to inhibit tumor angiogenesis. Additionally, Endostar has the ability to modulate signaling pathways, such as PI3K/PKB and Wnt/β-catenin, to further enhance its anti-tumor efficacy.^[14,15]^ Multiple clinical multicenter studies have shown that the combination of Endostar and chemotherapy has been proven to prolong overall survival, progression-free survival, and median tumor progression time in patients with advanced lung cancer. Additionally, it has been found to improve the quality of life for these patients.^[16,17]^

Although Endostar, whether used alone or in combination with other drugs, has a high safety profile, it is inevitable that Endostar also presents common adverse reactions associated with anti-angiogenic drugs, such as hypertension, bleeding, and proteinuria. Overall, the incidence of adverse reactions with Endostar is relatively lower compared to other drugs. A prospective study was conducted on a cohort of 73 patients diagnosed with stage III NSCLC, with 66% of them having lung squamous cell carcinoma. The study aimed to investigate the effectiveness of combining Endostar with concurrent chemoradiotherapy. Despite a significant proportion (25%) of the enrolled population having a documented history of cardiovascular disease, no adverse cardiovascular events were observed in relation to the administration of Endostar combination therapy.^[18]^

The clinical studies mentioned above regarding Endostar are consistent with our own observations during its practical application. The majority of patients have been found to safely use Endostar in combination with chemotherapy. The typical adverse cardiac reactions associated with Endostar are usually mild to moderate and reversible toxic responses, such as myocardial ischemia and arrhythmia. These reactions do not have a significant impact on the treatment process or the safety of patients’ lives.^[19]^ In contrast to previous studies that utilized Endostar in combination with chemotherapy, concurrent chemoradiotherapy, or targeted drugs to assess the safety and adverse reactions of Endostar, this patient case is unique. After completing the required examinations and ruling out any relevant contraindications to treatment, the patient suddenly experienced exacerbated symptoms of chest tightness while undergoing single agent Endostar treatment. After ruling out dyspnea caused by pulmonary and other factors, a CT examination confirmed the diagnosis of acute pericardial tamponade.

Acute pericardial tamponade can be caused by various factors, such as aortic dissection, trauma, and tumors.^[20]^ This condition leads to the rapid accumulation of pericardial effusion or blood, resulting in clinical symptoms primarily characterized by Beck triad.^[21]^ These symptoms include blocked blood circulation in the heart, damage to cardiomyocytes, impaired diastolic function of the heart, and increased venous pressure, which can lead to pulmonary congestion. At this time, patients may experience symptoms of dyspnea. It often presents as orthopnea and difficulty breathing. At the ASCO meeting in 2022, PYI Phyo Aung and colleagues conducted a retrospective analysis of 7105 patients with NSCLC who developed pericardial tamponade. The study concluded that pericardial tamponade increases the risk of acute respiratory failure and in-hospital mortality for these patients.^[22]^ In light of the potential risk of acute pericardial tamponade, it is crucial to promptly identify the cause of worsened dyspnea in the diagnosis and treatment of lung cancer patients to exclude the possibility of acute pericardial tamponade. Dyspnea caused by pericardial tamponade should first be differentiated from pulmonary dyspnea.^[23]^ This includes inspiratory dyspnea caused by large airway obstruction, expiratory dyspnea induced by bronchial asthma, and mixed dyspnea resulting from pleural lesions. These conditions can be easily identified through the patient medical history, PE, and lung CT scan. Secondly, it should be distinguished from acute coronary syndrome and cardiogenic dyspnea caused by heart failure.^[24]^ Patients with specific cardiopulmonary diseases, such as rheumatic heart disease and pulmonary heart disease, can be diagnosed using electrocardiograms, cardiac ultrasound Doppler, and blood tests. Finally, it is important to distinguish between toxic dyspnea caused by opioids and neurogenic dyspnea caused by intracranial tumors.^[25]^ Once acute pericardial tamponade occurs, immediate interventions such as pericardial puncture and drainage, pericardiotomy, and other treatments to alleviate cardiac compression should be implemented as soon as possible.^[26]^

The challenge in diagnosing acute pericardial tamponade in this patient with NSCLC is that the patient had recently undergone a CT examination before starting Endostar medication. Additionally, the patient has a history of asthma and previous episodes of stuffy symptoms, which could potentially mask the symptoms of acute pericardial tamponade. The Beck triad was atypical when the patient initially experienced worsening dyspnea. At that time, there was no significant downward trend in blood pressure, and there was no fever. This also led to our failure to promptly conduct a cardiac ultrasound or CT examination to assess the situation. After 7 days of Endostar treatment, we promptly confirmed the presence of acute pericardial tamponade using CT and immediately contacted the Department of Cardiology for consultation. The Department of Cardiology, recommends immediate pericardial puncture to stabilize the patient vital signs, considering the patient medical history and treatment process. This is due to the possibility of drug-related acute pericardial tamponade caused by Endostar, which cannot be ruled out. After discussing with his children, the patient believed that his condition was critical and decided to forgo pericardiocentesis and any further treatment. Our persuasion was ineffective, so the patient was automatically discharged on October 31. During the subsequent follow-up telephone call with the patient children, we discovered that the patient dyspnea had worsened upon returning home. He was unable to lie down and rest all day, and ultimately passed away at home 2 days after being discharged.

Previous safety-related research data on Endostar were primarily obtained from observations of combined chemotherapy.^[27]^ However, in this case, the patient experienced acute pericardial tamponade after using Endostar alone, without any other anti-tumor drugs. Although the direct causal relationship between the 2 cannot be directly confirmed, it also suggests an association between Endostar and acute pericardial tamponade. Although numerous prospective and retrospective studies have suggested that combined treatment with Endostar may lead to an increase in cardiovascular-related events, these events were only mild to moderate adverse reactions, and no serious adverse reactions were observed. Acute pericardial tamponade is a highly serious and potentially fatal complication. Although it can also be classified as a cardiovascular event, its level of risk is not equivalent to the level of attention we need to give it. What requires our attention is the fact that no scholars have previously reported similar complications. This may cause clinicians to overlook the possibility of complications associated with acute pericardial tamponade, despite the extremely low probability indicated by the current study.

The analysis above discusses the safety profile and the process of conducting differential diagnosis for Endostar, an endogenous angiogenesis inhibitor. Overall, Endostar offers several advantages as an enhanced version of the endogenous angiogenesis inhibitor. However, its drawbacks include high dosage requirements,^[28]^ a short half-life, and serum instability.^[29,30]^ These limitations may potentially be addressed by improving patient compliance through the use of devices such as infusion ports combined with infusion pumps, modifying the drug delivery methods, and optimizing the molecular structure, excipients, and drug delivery system of Endostar.

The price of Endostar is relatively high. In developing countries, where the number of NSCLC cases is increasing, the utilization of Endostar in conjunction with other medications or subsequent to radiotherapy might result in a decrease in the duration and total dosage of Endostar. This approach may be relatively feasible, balancing therapeutic efficacy with patient economic considerations.

### 3.1. Limitations

The acute and severe adverse cardiac reactions can be deadly. Although there is no clear report in the existing literature, clinicians should remain vigilant and consider the relevance of Endostar treatment. The drawback of this case report is that, being a descriptive retrospective study, it cannot directly establish a causal relationship between Endostar and acute pericardial tamponade. Further prospective studies are necessary to establish conclusive evidence. In the clinical treatment process, it is common to use Endostar concurrently with chemotherapy or targeted therapy agents. This polypharmacy approach complicates the task of isolating and precisely attributing cardiac events to Endostar. The presence of multiple therapeutic agents may confound the observed effects. The interaction of different drugs can complicate the precise cardiac safety profile of Endostar, requiring more analysis and careful monitoring to distinguish the actual frequency and characteristics of drug-related cardiac events.

## 4. Conclusion

Medical personnel should remain highly vigilant for the potential risk of Endostar inducing a severe and potentially lethal complication of acute pericardial tamponade, despite the extremely low probability based on previous data.

## Author contributions

**Data curation:** wanhui Dong, Qingming Sun, Dezhen Wu.

**Formal analysis:** wanhui Dong, Qingming Sun, Dezhen Wu.

**Investigation:** wanhui Dong, Qingming Sun, Dezhen Wu.

**Methodology:** Dezhen Wu.

**Software:** Pei Zhu.

**Supervision:** Mingxing Wang.

**Validation:** Mingxing Wang.

**Visualization:** Mingxing Wang, Pei Zhu.

**Writing – review & editing:** Wanhui Dong, Mingxing Wang, Pei Zhu.
